# rClone Red facilitates bacterial gene expression research by undergraduates in the teaching laboratory

**DOI:** 10.1093/synbio/ysy013

**Published:** 2018-08-07

**Authors:** A Malcolm Campbell, Todd T Eckdahl

**Affiliations:** 1 Biology Department, Davidson College, Davidson, NC, USA; 2 Martin Genomics Program, Davidson College, Davidson, NC, USA; 3 Biology Department, Missouri Western State University, Saint Joseph, MO, USA

**Keywords:** translation initiation, synthetic biology, course-based undergraduate research experience (CURE), golden gate assembly, ribosome binding site

## Abstract

rClone Red is a low-cost and student-friendly research tool that has been used successfully in undergraduate teaching laboratories. It enables students to perform original research within the financial and time constraints of a typical undergraduate environment. Students can strengthen their understanding of the initiation of bacterial translation by cloning ribosomal binding sites of their own design and using a red fluorescent protein reporter to measure translation efficiency. Online microbial genome sequences and the mFold website enable students to explore homologous rRNA gene sequences and RNA folding, respectively. In this report, we described how students in a genetics course who were given the opportunity to use rClone Red demonstrated significant learning gains on 16 of 20 concepts, and made original discoveries about the function of ribosome binding sites. By combining the highly successful cloning method of golden gate assembly with the dual reporter proteins of green fluorescent protein and red fluorescent protein, rClone Red enables novice undergraduates to make new discoveries about the mechanisms of translational initiation, while learning the core concepts of genetic information flow in bacteria.

## 1. Introduction

Education in STEM (science, technology, engineering and math) has reached an intersection where opportunities can meet challenges. We want our students to learn cutting edge methods but do so with meaningful learning outcomes. Students, teaching faculty, professional societies and the government have all called for better methods of hands-on learning that can improve student performance in core competencies (1, 2). Increasingly, STEM educators are turning to course-based undergraduate research experiences (CUREs) as a way to reach a larger number of students with diverse demographics, while containing expenditures (3–6). The redesigned Advanced Placement (AP) Biology course has urged teachers to provide laboratory experiences for high school students despite limitations associated with cost, facilities and teacher training (7). The Next Generation Science Standards (NGSS) also calls on high school biology teachers to provide students with hands-on learning opportunities with open-end laboratory experiences (8). The field of synthetic biology has grown rapidly to provide inexpensive, student-friendly lab-based options, some of which were developed by undergraduate students in the International Genetically Engineered Machines (iGEM) competition (9).

**Figure 1.**
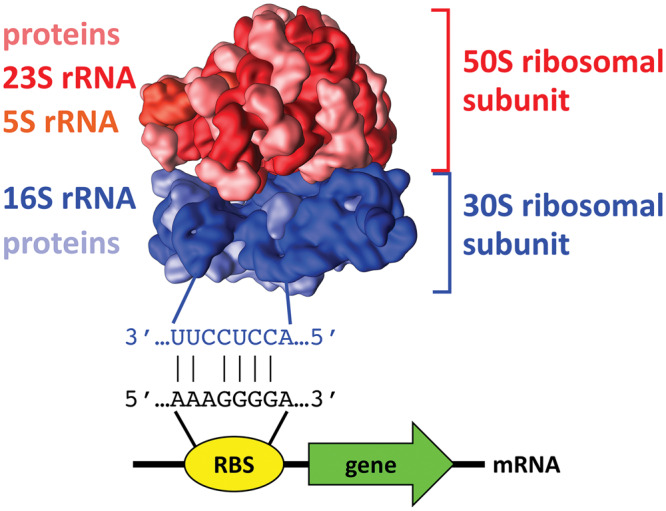
Structure and shape of the *E. coli* 70S ribosome. The large 50S ribosomal subunit (red) and small 30S ribosomal subunit (blue) are shown with their constituent parts. For the 50S subunit, the 23S (dark red) and 5S (orange red) rRNAs and the ribosomal proteins (pink) are shown. For the 30S subunit, the 16S rRNA (dark blue) and the ribosomal proteins (light blue) are shown. Ribosome image created by Vossman.

Students who use a traditional Genetics textbook to learn about protein-encoding gene expression initially focus on the flow of genetic information from DNA to RNA to proteins, according to the ‘central dogma’. Thereafter, they learn that transcription is regulated largely by promoters and nearby regulatory DNA elements located upstream of the protein-encoding coding DNA sequence (10). When students study translation, they usually focus on the protein coding sequence that begins with the start codon and ends with a stop codon. However, teachers and students alike often skip over a very important control element that regulates the initiation of translation. The ribosomal binding site (RBS) is a short segment of bacterial mRNA located upstream of the start codon (Figure 1). The mRNA sequence of the RBS is a key factor in determining the overall efficiency of translation because the 16S rRNA of the 30S ribosomal subunit must base pair with the mRNA in order for translation to begin. It would be especially helpful if students could conduct research on the RBS using cost-effective synthetic biology methods that are easy to master.

We developed pClone Red to allow students to conduct research on promoters (11). Even novice students are able to clone new promoters into pClone Red because it takes advantage of golden gate assembly (GGA) cloning (12, 13). GGA uses type IIs restriction enzymes that cut outside their asymmetric recognition sequences and produce sticky ends that can be selected by users. We have found that first year college students and high school students are remarkably successful when using GGA to clone novel promoters and carry out mutational analysis of existing promoters from synthetic oligonucleotides. In previous education publications, we described our use of pClone Red to help undergraduates better understand the regulation of the initiation of transcription by investigating promoter sequences (14, 15). In this education research, we describe how rClone Red can be used by students to design and test RBSs. Previously, we described rClone Red in a basic research publication (16). Independent research students working outside laboratory courses conducted research to examine different RBS designs and ‘anti-RBS’ sequences within the mRNA that reduced reporter protein production. The current education study explains how faculty can foster student understanding, while adhering to typical laboratory course budgets and schedule constraints. We explain how the design of rClone Red makes it easy for early career STEM students to identify clones that contain the RBS they designed, and to measure RBS strengths with a reporter gene. We used pre-/post-assessment to measure, which concepts students learned from a laboratory-based curriculum that included rClone Red.

## 2. Materials and methods

### 2.1 Cloning methods

For use in cloning with rClone Red, students design two oligonucleotides between 15 and 60 bases long that are ordered from Integrated DNA Technologies, Inc. as ‘lab ready’ solutions of 100 µM each. Students have access to a free online tool we developed called Oligator (http://gcat.davidson.edu/iGem10/index.html) that reduces errors in sequence design. In the final design, the top strand sequence must begin with the left sticky end of 5′ CGAC 3′, and the bottom strand must begin with the right sticky end of 5′ CCGC 3′. The Oligator ensures that neither strand contains a Bsa I recognition site. Oligos are annealed to form double-stranded DNA using a simple overnight protocol. At least one day before lab, one student from each group prepares an annealing solution that contains 100 mM NaCl, 10 mM Tris-HCl, pH 7.4 and both oligonucleotides at 5 µM in a final volume of 20 µL. Tubes (500 µl) with the mixed oligonucleotides are floated for 10 min in a beaker containing 600 ml of boiling water, after which time the heat is turned off and the mixed oligonucleotides are allowed to cool slowly and anneal in the water bath overnight. Annealed oligonucleotides are used the next day or stored frozen at −20°C and used later.

GGA combines BsaI-HF v2 type IIs restriction enzyme (New England Biolabs) and T4 DNA ligase (Promega) as described (12, 13). The plasmid rClone Red (J100272; Genbank accession number MH549194) produces GFP when unaltered because it has a ‘left-facing’ promoter, RBS and GFP ORF, all of which is flanked by two BsaI restriction sites. To the left of this backwards GFP expression cassette is a ‘right facing’ promoter. To the right of the GFP cassette is a RFP ORF. When GGA is performed, the GFP cassette is removed and a new RBS encoded by students’ annealed oligonucleotides is cloned between the right-facing promoter and the RFP ORF, producing a new RFP expression cassette. Each GGA reaction contains 50 ng of rClone Red plasmid, 4 nM annealed oligonucleotides, 0.5 µl BsaI HFv2, 0.5 µl ligase and 1X ligase buffer in a final volume of 10 µl. A thermal cycler is used to perform 30 cycles of 1 min at 37°C followed by 1 min at 16°C. A final 5 min incubation at 37°C reduces the frequency of background green colonies. Competent JM109 *Escherichia coli* (Zymo Research) are transformed following the manufacturers recommendations, and transformed cells are plated on LB ampicillin (50 µg/ml) agar plates. Carolina Biological sells an rClone Red kit that contains all reagents, including a positive control RBS, competent cells and antibiotic-containing agar plates. Student-designed RBSs must be purchased separately.

For each RBS tested, three non-green candidate colonies are picked with a sterile toothpick and grown in shaking incubator overnight at 37°C in 4 ml of LB ampicillin (50 µg/ml) liquid media. The next day, 200 µl are placed in 96 well plates for quantification of RFP fluorescence intensity with a BioTek Synergy H1 microplate reader. RFP was measured in arbitrary fluorescence units using 585 nm for excitation and 615 nm for emission, and the concentration of bacteria was measured using optical density at 590 nM. Students calculate the ratio of fluorescence divided by absorbance to normalize for cell culture density. Students convert their raw fluorescence values into normalized values because not all cultures will have the same density of cells.

For two-dimensional RNA folding analysis, students use Mfold, a free online tool (17). Students can submit their RBS designs to Mfold to predict the stability of any secondary RNA structures that might prevent the initiation of translation. Mfold reports the stability of several possible RNA folding structures with Δ*G*, where larger negative Δ*G* values indicate more stable structures.

Photographs of colonies from rClone experiments are taken by normal digital cameras and not fluorescence microscopes or dissecting scopes. Our students routinely use their cell phones to capture images similar to the ones in this report for use in their oral presentations and laboratory reports.

### 2.2 Educational methods

The genetics course for this educational research project was taught at Missouri Western State University (MWSU), a primarily undergraduate institution with open admissions. Most MWSU students live within a 50 mile radius and many are first generation college students, which means they are the first in their families to attend college. The genetics course is required for Biology majors and most students enrolled in the course are sophomores. The course included three 50 min lecture/discussion periods and one 3 h laboratory session per week. The prerequisites for the course are one semester of introductory cellular biology and one semester of general chemistry. There were 50 students enrolled in the course, which was divided into three laboratory sections. One of us (T.T.E.) was the sole instructor for all lecture and laboratory sections of the course. Four weeks of laboratory sessions were dedicated to an rClone Red laboratory module that complemented lecture material from the course. Prior to the laboratory module, students had already learned to pipet small volumes of liquid and work sterilely with bacteria, and they had learned about DNA base pairing, transcription and translation.

To assess learning gains among undergraduates using rClone Red, we developed a 20 question multiple choice assessment instrument (available to instructors upon request). In the MWSU genetics course, the instrument was administered to 50 MWSU genetics students at the beginning of the fall 2015 semester and again at the end of the semester to 48 students (one week after the rClone Red module had been completed). IRB exemption #2416 was granted prior to the study. Significant differences between pre- and post-survey scores in Figure 4 were determined using an unpaired *z* test for difference in proportion. The assessment data in this study were collected in the fall of 2015, but rClone Red has been used repeatedly in subsequent semesters at MWSU and at Davidson College. There is only one Genetics class each semester at MWSU, so it was not possible to have a control group of students taking Genetics that was not using rClone Red.

## 3. Results

### 3.1 Biological context

**Figure 2.**
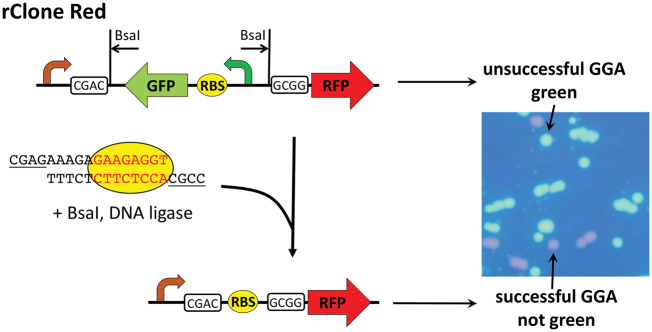
rClone Red design and use. Unsuccessful GGA using rClone Red results in colonies that express GFP, as indicated. Successful GGA with a new RBS (yellow oval) results from ligation using the CGAC left end and GCGG right sticky ends (underlined). Colonies carrying a new RBS do not express GFP, but express RFP at a level determined by the strength of the new RBS.

Our goal in developing rClone Red was to offer students a CURE without having to reconfigure our normal laboratory schedules or exceeding our existing budgets for laboratory supplies. rClone Red empowers undergraduates to conduct original research on the control of translation initiation without the allocation of additional time or funding. The simplicity and efficiency of GGA makes rClone Red appropriate for teaching laboratory courses with novice students. The design of rClone Red enables students to easily determine, which colonies in a cloning experiment have successfully incorporated a new RBS (Figure 2). Bacteria that have been transformed with the original rClone Red plasmid express GFP using the left-facing expression cassette, and are easily visible on a UV or blue light box as bright green colonies. Because GGA replaces the backwards GFP expression cassette with a new RBS, colonies resulting from successful GGA are not green. Our students who are color blind learned to detect the difference between fluorescing green and non-fluorescent colonies. The extent to which successful colonies glow red from the resulting RFP reporter gene using the right-facing promoter and the new RBS depends on the ability of the new RBS to initiate translation. The colonies in Figure 2 are from a strong RBS, so the colonies appear bright red. Once they are shown the differences in control GFP and RFP plates, color blind students learned to detect the difference between GFP and RFP because GFP is so much brighter than RFP (see Figure 2).

**Figure 3.**
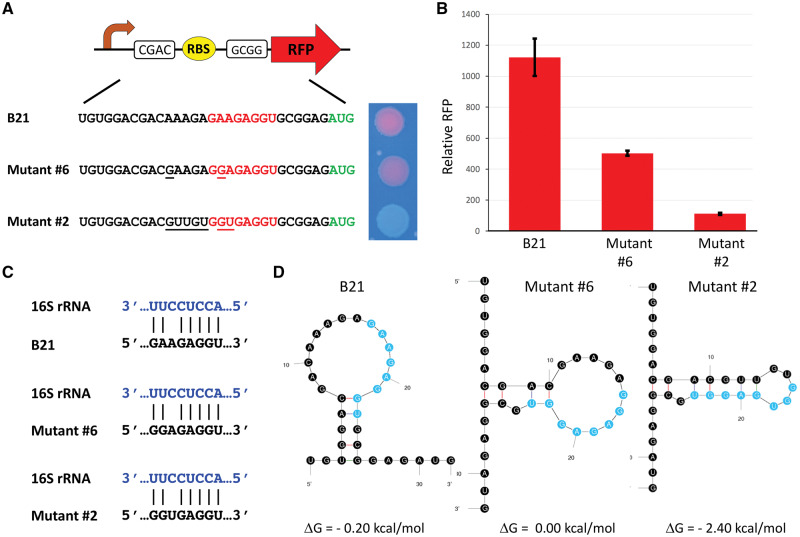
Examples of student rClone Red results. (**A**) Sequences of the original B21 RBS, mutant #6 and mutant #2, including flanking bases, RBSs (red) and the RFP start codon (green). The photograph shows all three clones spotted on a plate. Mutations in #6 and #2 compared with B21 are underlined. (**B**) Relative RFP (red fluorescence divided by absorbance at 590 nM) average ± SEM for B21, mutant #6 and mutant #2. (**C**) Base paring between B21, mutant #6 and mutant #2 and 16S rRNA. (**D**) Most stable structures predicted by mFold for B21, mutant #6 and mutant #2.

Genetics students at MWSU learned in lecture about the basic mechanisms by which RBSs initiate translation and were given the opportunity to enhance their learning by conducting original research by participating in an rClone Red CURE. They began their laboratory investigation by examining the sequence of a strong RBS used as a positive control that produced red colonies (Figure 3A, top). The genetics students proposed new mutations that would enable them to explore the sequence requirements for RBS function. The strong RBS (B21) is composed of eight bases, but the oligonucleotide students used also contained flanking bases and sticky ends to produce directional ligation upstream of the RFP start codon in rClone Red. One group of students designed mutant #6 which differs from B21 by one base upstream of the RBS and one base in the RBS itself (see Figure 3A, middle). Another group designed mutant #2, which has five mutations upstream of the RBS and two mutations in the RBS (see Figure 3A, bottom). The photograph of 2 µl drops of cultures on a plate shows that mutant #6 does not produce as much red fluorescence as B21, and mutant #2 does not produce any visible RFP. Because color perception is influenced by genetics (18), students used a fluorometer to quantify fluorescence intensity as an indirect measure of translation efficiency (Figure 3B). When the students tried to understand their results, they were confused initially because mutants #6 and #2 base pair with the 16S rRNA as well as the strong RBS B21 (Figure 3C). For all three RBSs, seven of the eight bases form hydrogen bonds with the 16S rRNA of the small ribosomal subunit. The students expanded their understanding of RBS secondary structure when they used the online RNA folding tool Mfold (Figure 3D) (17, 19, 20). Both B21 and mutant #6 are predicted to form loop structures that are anchored by a four base pair stem, including two bases of the RBS. In contrast, the predicted structure for mutant #2 contains a six base pair stem including four bases of the RBS. These RNA folding predictions allowed the students to propose a structural explanation why the more energetically stable mutant #2 (ΔG = −2.40 kcal/mol) produced a weaker RBS than B21 or mutant #6, which are energetically less stable and have more bases available for pairing to the 16S rRNA.

### 3.2 Educational outcomes

**Figure 4.**
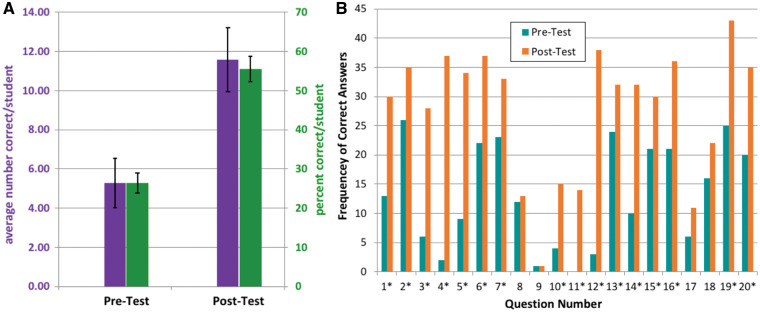
Students made significant learning gains. (**A**) Students performed better on the assessment after conducting their rClone research projects. Purple bars (left *y*-axis) indicate the average number of correct question out of 20 possible (5.28 pre-test; 11.58 post-test; ±SEM). Green bars (right *y*-axis) indicate percent correct (26.4 pre-test; 55.6 post-test; ±SEM). (**B**) Total number of students with the correct answer for each question before the lab (pre-test, teal) and after the lab (post-test, orange). Numbers match learning objectives in *Table 1*, and asterisk (*) denotes significant differences pre-test versus post-test, *P* < 0.05.

Before we implemented rClone Red in the Genetics teaching laboratory, we developed a list of 20 concepts that we anticipated students might learn as a result of their laboratory research (Table 1). All 20 concepts are addressed by the instructor to varying degrees, while students are working in the lab, but we wanted to know which ones would stick with the students and which ones would not. We developed a 20 question multiple choice assessment instrument to determine what students knew coming into the class (pre-test) and what they knew 1 week after the rClone Red CURE was completed (post-test; Figure 4A). On average, students correctly answered 5.28 questions on the 20 question pre-test (26.4%). After completing their RBS research projects, students correctly answered an average of 11.6 out of 20 questions (55.6%).

**Table 1.** Twenty concepts addressed during rClone Red research projects that students might learn

1. Initiation of translation—ribosome interacts with RBS and start codon	11. Annealing oligonucleotides to build RBS
2. Gene regulation at translational level—RBS efficiency	12. rClone system—green versus not green
3. Interaction between RBS and 16S rRNA	13. Reporter genes—RFP or Blue chromoprotein, GFP negative control
4. Base pairing in RNA—alternatives to Watson/Crick	14. RBS strengths—intensity of reporter gene signal, red or blue
5. Abstraction and levels of hierarchy in Synthetic Biology—DNA, parts, devices, systems	15. Mutagenesis as an approach to understanding function
6. Standardization of parts in Synthetic Biology—Registry of Standard Biological Parts	16. Consensus sequences used in molecular biology
7. Standards of assembly in Synthetic Biology	17. Choose RBSs to control protein production amplitude
8. Golden gate assembly method	18. RBSs with various efficiencies within bacterial genomes
9. Type IIs restriction enzymes—directional cutting, sticky ends chosen	19. RBS efficiency as a natural contributor to phenotype
10. Design oligonucleotides for dsDNA	20. RBS efficiency acted upon by natural selection

Because average scores for the overall 20 question assessment obscured which topics students learned, we examined each of the 20 concepts individually and determined which ones showed significant learning gains and which ones did not (Figure 4B). Students showed significant improvements for 16 of the 20 concepts. The four concepts that students did not learn were: #8, the GGA method; #9, characteristics of type IIs restriction enzymes; #17, altering RBS strength to regulate protein production level; and #18, selective advantage for bacteria to have different strength RBSs. We had hypothesized that students would acquire all 20 concepts equally well, but the assessment demonstrated that either the students did not learn these four, or our ability to measure these four was imperfect. Regardless of the reason, we can revise our teaching and assessment approach for these four concepts if we feel they are important enough to do so. Only through assessment can we know which portions of our curriculum works, and what needs improvement.

## 4. Discussion

Since 2015, we have been using rClone Red for teaching Genetics at MWSU and Introductory Biology for majors at Davidson College. rClone Red is a powerful tool that enables any student to have a classroom-based research experience (CURE) (3–6). Every biology student is taught the core concept of central dogma and our impression from other faculty is that transcriptional regulation is often emphasized more than translational regulation. Some students are given opportunities to explore experimentally the mechanisms by which transcription is initiated and regulated, but it seems that very few get the chance to study the initiation and regulation of translation. The initiation of bacterial translation involves base pairing between the mRNA and the 16S rRNA of the small subunit of the ribosome (Figure 1). However, scientists have a limited understanding of the roles of bases flanking the RBS and RNA secondary structure, which creates opportunities for students to make new discoveries. Before modification, rClone Red expresses GFP so that students can easily detect which colonies contain the original plasmid. Cloning by GGA works nearly 100% of the time even with novice students. Beginners quickly realize that non-green colonies contain GGA products in which the GFP expression cassette was successfully replaced with their RBS (see Figure 2). The intensity of red fluorescence is determined by the strength of the RBS, which is influenced by rRNA base pairing and the three-dimensional folding of the mRNA (Figure 3) (19, 20). Students conducted RBS mutational analyses, collected original data on the strengths of mutant RBSs, and provided explanations for their results using Mfold (see Figure 3) (17). RNA folding can be explored further by using the online RBS calculator tool, which predicts RBS strength (20).

Many teaching laboratories do not have access to a fluorescence microscope to photograph colonies, or a fluorometer to quantify fluorescence intensity. However, cell phones are capable of producing the images shown in this report, as long as a UV or blue light source is available. We reported previously that cell phone images can be used in conjunction with the free software ImageJ to compare relative RFP intensities (11, 21). rClone Red can be used anywhere there is a UV or blue light source, cell phones and computers with ImageJ installed. Instructors who do not feel comfortable teaching ImageJ could rely upon visual perception by students to rank color intensity, though color-blind students would require assistance from peers.

In this education research project with genetics students at MWSU, we found that students learned most of the 20 concepts that we had identified prior to using rClone Red (Table 1 and Figure 4). It would have been nice to compare students in the rClone Red lab to an equivalent population not using rClone Red, but there is only one Genetics class each semester at MWSU, and it is taught by the same instructor each time. It would be interesting to have faculty at larger campuses conduct similar rClone Red educational research along with a control population. One of us (A.M.C.) is conducting additional education research using rClone Red as well as pClone Red to measure self-efficacy, self-identity and project ownership by students in a CURE setting.

When pClone was used with genetics and introductory biology students on both campuses, there were some learning outcome differences between the populations (11). For example, the differences between type IIs and type II restriction enzymes was better understood by the Genetics students. Conversely, GGA was better understood by the introductory students. These learning outcome differences are intriguing and may indicate that beginning students are more amenable to learning a new way of cloning if they have not learned a traditional cloning method in a previous course. Genetic students might be better prepared to distinguish the two types of restriction enzymes due to prior experience with restriction enzymes in general. In this educational study using rClone Red, the MWSU genetics students failed to improve their understanding of GGA similar to previous MWSU genetics students had when using pClone Red. Instruction was altered during the genetics laboratory sessions to review GGA each lab and yet this concept did not stick well with the students. Further investigation is required before we can determine the best way to help genetics students learn GGA as a new and alternative way to clone DNA.

Many education scholars have studied the value of multiple choice questions (22–25). From the literature, it is clear that multiple choice questions can reveal student learning outcomes. Our results show that students learned some concepts but not others because students improved their scores on several concepts but not all of them. Higher order thinking skills are difficult to assess with multiple choice questions, which is why MWSU students also must submit laboratory reports and give oral presentations for their genetics course grades.

In addition to its utility for CUREs, the simple elegance of the rClone Red design also makes it amendable for undergraduates or high schoolers who want to have summer undergraduate research experiences (SUREs) (16, 26). There are several interesting areas of investigation that students could explore with rClone Red. Because *E. coli* has more than one 16S rRNA gene, students could design RBS sequences that target one or more of the seven rRNA paralogous operons (27, 28). Students could generate libraries of potential RBS sequences by inserting 1-8 N bases during oligonucleotide synthesis and selecting RBS strengths for specific desired applications (16). Another application of rClone Red is using it for testing the specificity of a riboswitch and its potential for translational actuation (29). Riboswitches are RNA biosensors incorporated into the 5′ end of mRNAs that use ligand-dependent RNA folding to control gene expression at the levels of transcription or translation (30). Students could use rClone Red to investigate the functions of natural and synthetic riboswitches or engineer new ones.

rClone Red is one member of a family of plasmids we have developed to blur the lines between teaching and research laboratories. In addition to pClone Red (11), we have designed and built tClone Red, actClone Red and repClone Red. Each of these plasmids uses GGA, a GFP reporter gene that is silenced when students successfully clone their new DNA part, and a RFP reporter to measure gene expression. tClone Red (http://parts.igem.org/Part: BBa_J119361) supports the investigation of rho-independent transcriptional terminators and riboswitches that use alternative RNA folding states to control gene expression. tClone Red has been used successfully in the genetics teaching laboratory at MWSU and in independent undergraduate research projects on both of our campuses. actClone Red (http://parts.igem.org/wiki/index.php/Part:BBa_J100435) enables students to reengineer the *E. coli ompC* promoter, which requires three endogenous ompR proteins for activation. Student can change the order and/or sequence of the ompR binding sites to test how malleable the promoter is to sequence perturbations. repClone Red (http://parts.igem.org/wiki/index.php/Part:BBa_J100342) is built for the pTet promoter and the tetR repressor protein. Students can alter the sequence and order of two tetR binding sites to explore this interesting promoter. actClone Red and repClone Red have been used in teaching laboratories at Davidson College, but they are unstable constructs and suffer from unwanted recombination events. We are working to improve their designs so that they are more student-friendly.

rClone Red is a cost-effective educational tool for use in teaching laboratory courses that allow students to conduct original research within typical laboratory course time constraints and budget limitations. It has been used with sophomore genetics students as well as first year introductory biology students. rClone Red is appropriate for any laboratory course where the central dogma or bioengineering are included in the learning goals. We have shown that students learn many of the concepts we hoped they would, but there is room for improvement for a subset of these concepts. Additional education research by others, with a control population, would enhance our understanding of how rClone Red improves student understanding while simultaneously providing them with a CURE.
